# Vaccine Prices: A Systematic Review of Literature

**DOI:** 10.3390/vaccines8040629

**Published:** 2020-10-29

**Authors:** Rabia Hussain, Nadeem Irfan Bukhari, Anees ur Rehman, Mohamed Azmi Hassali, Zaheer-Ud-Din Babar

**Affiliations:** 1 Commonwealth Pharmacists Association, London E1W 1AW, UK; rabia.hussain2010@gmail.com; 2 Faculty of Pharmacy, The University of Lahore, Lahore 54590, Pakistan; 3 Punjab University College of Pharmacy, University of the Punjab, Allama Iqbal Campus, Lahore 54000, Pakistan; nadeem_irfan@hotmail.com; 4 Department of Clinical Pharmacy, School of Pharmaceutical Sciences, Universiti Sains Malaysia, Penang 11800, Malaysia; aneesurrehmanr90@gmail.com (A.u.R.); azmihassali@gmail.com (M.A.H.); 5 Department of Social and Administrative Pharmacy, School of Pharmaceutical Sciences, Universiti Sains Malaysia, Penang 11800, Malaysia; 6 Department of Pharmacy, University of Huddersfield, Huddersfield, Queensgate HD1 3DH, UK

**Keywords:** vaccines, prices, GAVI, human papilloma vaccine, extended program on immunization, childhood vaccines, UNICEF

## Abstract

Vaccines are among the most vital interventions to control and reduce the morbidity and mortality worldwide. In accessing vaccines, pricing is usually the single most important deciding element. However, there is a scarcity of the literature on the vaccines pricing. The current study aims to review vaccine prices from the published literature and to evaluate factors that impact the pricing of vaccines. The literature (from 2015–2020) was reviewed to identify the original research articles. Systematic searches were conducted across the five databases including, Google Scholar, PubMed, Science Direct, Scopus and Springer Link. Literature search yielded 23,626 articles, of which 7351 were screened and 7310 articles were excluded based on title and abstracts relevance. The 41 studies were selected for full text review and 4 studies were found to meet the inclusion criteria. The included studies discussed vaccine prices for childhood vaccines, for Human Papilloma Virus (HPV) in US, China and in Europe. One study detailed the various scenarios of the HPV vaccines pricing. It was found that recently introduced vaccines have higher prices owing to the involvement of technology and research for their manufacture. However, prices tended to decrease over some maturation in price and by the involvement of Global Alliance for Vaccine Initiative (GAVI) and other allies. The prices of vaccines in China were much lower than the other high-income countries and the prices offered through United Nations Children’s Fund (UNICEF), mainly due to the large scale of demand in China. The affordable prices of vaccines were related to delicate procedures involving multiple stakeholders and a shorter duration of contract. This review systematically evaluated the literature and identified key factors that could impact vaccines pricing. The prices were higher for the newly introduced vaccines into the market. However, with the price maturation, there was a decline in the pricing and affordable prices could be achieved through tender pricing and involvement of GAVI and other allies.

## 1. Introduction

Vaccines are crucial to alleviate human sufferings and to reduce the avoidable deaths. The World Health Organization (WHO) has estimated that vaccines save 2–3 million lives each year globally [1]. The Global Vaccine Action Plan (GVAP) prevents millions of deaths through more equitable access to existing vaccines in all communities by 2020. To achieve the GVAP goals, the GVAP involves the leadership of the Bill & Melinda Gates Foundation, Global Alliance for Vaccine Initiative (GAVI) Alliance, World Health Organization (WHO), United States National Institute of Allergies and Infectious Diseases, United Nations Children’s Fund (UNICEF), health professionals, academia, manufacturers, global agencies, governments and elected officials, civil society, media and the private sector [2,3,4]

The Expanded Program on Immunization (EPI) was established in 1974 and has proved to be an important initiative. As a result, to date, the immunization coverage has increased from 5% to 86% and in some countries reaching up to 95% [5]. In recent years, the price transparency in vaccines is being emphasized more than any other factor as the vaccines pricing has lesser transparency than the pricing of any other lifesaving pharmaceuticals [6].

Based on the procurement, the global market share of vaccines can be divided into the following main segments: (1) countries procuring through UNICEF, eligible for GAVI support (2) middle-income countries procuring through UNICEF but not eligible for GAVI support, (3) countries procuring through Pan American Health Organization Revolving Fund (PAHO-RF), (4) middle-income countries that self-procure all or the most of vaccines and (5) self-procuring high-income countries [7]. The price and timely access to vaccines is majorly affected by the different procurement mechanisms. Such as the countries that procure their vaccines directly from the suppliers has complete autonomy over vaccine pricing, selection and delivery process. Those involving UNICEF and PAHO-RF receive the benefits through large procurement pools [8].

The pricing of a new vaccine is a complex process that could have significant long-standing scientific, medical and public health implications. Pricing may have a major effect on new vaccine acceptance and, thus, either culminate or disrupt years of research and development and public health efforts [9]. Pricing strategy consists of many characteristics including population analysis, mapping of potential competitors, constructing a vaccine target product profile and estimating vaccine price-demand curve. It also involves the calculation of vaccine costs such as manufacturing, distribution, research and development costs, regulatory factors, pricing objectives and pricing structure [9]. Recently, few manufacturers have started to publicize the criteria used in the pricing process, however none of them report all the specific prices in all markets [6].

In low- and middle-income countries (LMICs), where vaccines are heavily subsidized to keep prices manageable [10], it is worth considering which stakeholder would be responsible to keep the prices low. Whether these are vaccine manufacturers, non-governmental organizations, private foundations or a combination of the above stakeholders. The other questions could be whether the vaccine prices be determined by the development costs or by the value they possess. One can also argue that if the value provided by a vaccine cannot recoup the cost of development over some reasonable time horizon, then is there a place for such products? The answer may be either yes or no, depending on the metrics used to define the value (e.g., healthcare costs avoided, morbidity reduction, humanitarian factors) [11]. Determining an appropriate price of vaccine from a healthcare provider and government public perspective is an important issue. This also influences whether a vaccine manufacturer decides to remain in the market or otherwise [12]. 

However, literature is scant on pricing and factors involved in the fluctuation of vaccine prices. In this context, the present systematic review aims to explore original research published regarding vaccine prices. This would help to explore the factors and how these metrics could then be used to build evidence-informed policies in this context.

## 2. Materials and Methods 

### 2.1. Literature Search

The Preferred Reporting Items for Systematic Reviews and Meta-Analyses (PRISMA) guidelines for conducting systematic reviews were followed [13]. This study used systematic searches between April 2020 and May 2020 to identify peer-reviewed original research articles published between January 2015 and May 2020. The following databases were searched: PubMed, Google Scholar, Scopus, Science Direct, and Springer Link. To ensure the completeness of search the following relevant journals were also searched: Vaccine, The Lancet and PLOS One.

### 2.2. Search Strategy

**Figure 1:**
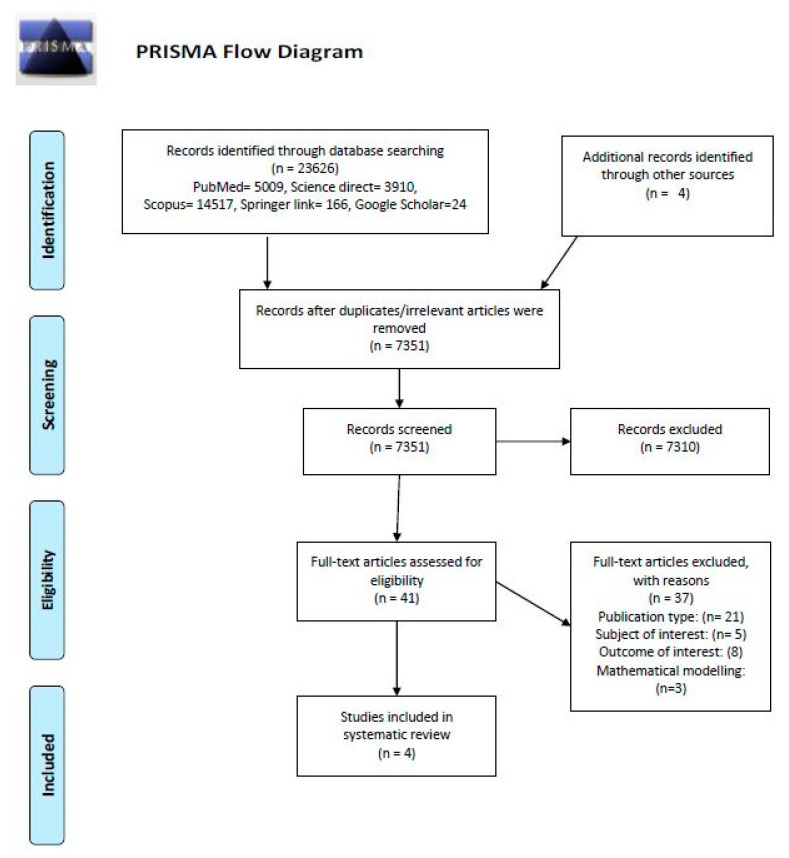
Preferred Reporting Items for Systematic Reviews and Meta-Analyses (PRISMA) flow diagram for the screening and selection of studies.

Both mapped and un-mapped terms were included for search strategy, which were illustrated in Table 1. The following keywords were included: (“Vaccine” or “Vaccines”) and (“Price” or “Pricing” or “Prices”). The keywords were combined and integrated in database and journal searches. “Boolean Operator” rules were utilized, and the terms used were searched using “AND” to combine the keywords and using “OR” to remove search duplication, where possible (Table 1). References lists of the retrieved articles were also assessed for relevant articles that may have missed during search process. The process of identification, screening, and inclusion of papers for this review is detailed in PRISMA format in Figure 1. 

### 2.3. Inclusion and Exclusion Criteria

Two reviewers independently screened the titles and abstracts, and selected the studies based on pre-defined inclusion and exclusion criteria. The studies were only included, if they were: (i) original research reporting the prices of vaccines, (ii) available as full text, (iii) published between Jan 2015 and May 2020 and published in English language. 

Studies evaluating the vaccine prices through mathematical modeling, commentaries, review articles, letters to editors or abstracts presented at scientific conferences were not included in this systematic review.

### 2.4. Quality Assessment 

To avoid bias in the study, selection of the articles was made considering the explicit method. The method recommends following the inclusion criteria strictly during search process to provide reliable data [14]. Any conflict between the two reviewers was resolved through discussion and consensus was developed.

### 2.5. Data Extraction

A data extraction sheet was developed. The items were finalized after discussion with the research team (RH, ZB, AR) and filled into the data extraction sheet. The information included authors, year, objectives, key findings, country, vaccine program, types of vaccine and vaccine prices. Data were extracted from eligible full text articles by two independent researchers (Table 2 and Table 3).

### 2.6. Outcome

The main outcome of the review was to assess the pricing of vaccines and the associated factors which could impact on pricing of vaccines.

## 3. Results

The literature search yielded 23,626 titles (PubMed = 5009, Science Direct = 3910, Scopus = 14,517, Springer Link = 166, Google Scholar = 24) through a database search. After the removal of irrelevant and duplicate records, 7351 abstracts were screened. Of those remaining, 41 articles were assessed as the full-length publications. The final four studies meeting the eligibility criteria were included in this study.

The data from the articles generated the following main themes.

1. Pricing of childhood vaccines
2. Pricing of Human Papilloma Virus (HPV) vaccines
3. Factors affecting the pricing of vaccines

### 3.1. Pricing of Childhood Vaccines

A United States (US)-based study analyzed prices for non influenza vaccines for children aged 0 to 18 years that were purchased by the public sector Vaccine For Children (VFC) program in the US. The mean vaccine price during 1996 to 2014 was about USD 11 for price-capped vaccines and USD 41 for combination vaccines (in 2014, excluding federal excise tax). The mean price of non-price-capped and non-combination vaccines was between USD 11 and USD 41 [15]. 

Another study from China also compared the procurement prices of Category 1 and 2 vaccines from China with those of Europe, US and UNICEF. This study was based on the median price for each vaccine. Category 1 vaccines were procured by the government and provided for the immunization free of cost, but for Category 2 vaccines, the parents have to pay out of the pocket for the immunization. The price range for Category 1 vaccines was USD 0.1–5.7, and for the Category 2 vaccines was USD 2.4–102.9. The procurement prices of Expanded Program on Immunization (EPI)-substitute vaccines (Category 2 vaccines against diseases preventable by Category 1 vaccines) were higher than that for their EPI equivalents. Category 1 vaccine prices were lower than that of the same vaccines in the US and European countries but were similar to UNICEF prices. The prices of domestically produced vaccines in China; Category 2 vaccines, such as seasonal influenza, Hemophilus influenza type b (Hib), Varicella (Var), and Oral Rabies Vaccine (ORV), were lower than that of the prices of similar vaccines in the US, Europe, and UNICEF. The prices of Pneumococcal conjugate vaccine (PCV7) and Diphtheria, Tetanus, Pertussis-Inactivated Polio vaccine/Hemophilus influenza type b (DTaP-IPV/Hib) were higher than that of the similar vaccine prices in the US, Europe and procured through UNICEF [16].

### 3.2. Pricing of Human Papilloma Virus (HPV) Vaccines

Herlihy et al. analyzed the pricing of Human Papilloma Vaccine (HPV) vaccines by considering six prices scenarios: including: (1) retail price, (2) after 37% tender price reduction, (3) fixed price of USD 8.50 per dose in upper-middle- and lower-middle-income countries, (4) subsidized prices from high-income countries, (5) subsidized price from manufacturer and (6) without tiered pricing. It was observed that the total HPV vaccine sales for the manufacturers of both bivalent and quadrivalent vaccines was USD 14.1 billion in 2006–14 counting a total fivefold return on investment to the manufacturers. It was also noted that with some maturation in price, there was a decrease in price, such as the prices of HPV vaccine funded by PAHO-RF decreased from USD 32.00 per dose in 2010 to USD 13.50 in 2013 and then to USD 8.50 in 2015 [17].

Similarly, Qendri et al. has retrieved the procurement notices and awards from 15 European countries, and found that prices of first-generation HPV vaccines decreased from USD 122.2 (EUR 101.8) in 2007 to USD 34.1 (EUR 28.4) in 2017, whereas the average dose price of the nine-valent vaccine in 2016–2017 was USD 58.9 (EUR 49.1). The unit prices were, respectively, in the range of USD 9.0 (EUR 7.5) and USD 41.3 (EUR 34.4) higher for the four-valent and nine-valent vaccines than for the two-valent vaccine [18].

(Exchange rate from Euro to USD in 2017: EUR 1 = USD 1.2011).

### 3.3. Factors Affecting the Pricing of Vaccines

The high prices were observed for multi components vaccines. However, the prices were found to be lower for combination vaccines [15]. Zheng et al. observed that prices of Category 1 vaccines in China were lower than many high-income countries and some UNICEF vaccine prices. The lower prices were achieved through the large-scale demand. Moreover, the higher prices of Category 2 vaccines were related to the limited demand and hence could not attain the economy of scale. Thus, manufacturers of Category 2 vaccines profit from the high prices and this further helps provide the manufacturers with the incentives to stay in the market with further investments for the research and development of the novel vaccines [16].

Qendri et al. observed that affordable prices of vaccines can be achieved through tender procedures as vaccine pricing is affected by contract volume, duration, country, per capita Gross Domestic Product (GDP) and number of offers received. On an annual basis, the contract volume was associated with a decrease in the per-dose price of USD 13.21 (EUR 11.0) per 100,000 doses per year. Additionally, regional level procurement of vaccines resulted in higher unit prices, USD 10.68 (EUR 8.9) per dose when compared to the procurement at the national level [18]. Similarly, offers received from both manufacturers lowered the unit price of vaccine by USD 5.40 (EUR 4.5) compared to procurement that received only one offer [18].

## 4. Discussion

This review provides a systematic search of literature between 2015 and 2020 of vaccines pricing and the factors affecting prices of vaccines. The result synthesis elaborates childhood and adult vaccines from China, US and Europe perspectives, thus adding to the current literature on the topic. In recent years, no other review on vaccines pricing has been performed so far. As a result of this analysis, different prices ranges were observed for the vaccines. It also analyzed the type of vaccines as well as factors affecting the prices of those vaccine. 

About 80% of the vaccines are produced by five big manufacturers including Glaxo Smith Kline, Merck, Novartis, Pfizer and Sanofi Pasteur [19,20]. In the past, vaccines were often considered as the less profitable business but due to change in global vaccine demand, the global vaccine market has increased [21,22].

It was also observed that more recently introduced childhood vaccines have higher prices, primarily due to the use of new and better technologies in vaccine development and production. Furthermore, as the manufacturers recoup the fixed cost of producing the vaccines, their prices are likely to go down over time because of the smaller marginal production cost. This could be ascribed to fact that many vaccines are only provided by one or two suppliers. New vaccines have particularly few suppliers because big investment is needed to develop a new vaccine [21]. 

A report from the WHO has also indicated that the vaccine prices are reduced when a vaccine is produced by a large number of suppliers (manufacturers) [6]. Another factor that leads to high prices is patent protection, as in the case of new vaccines, which tend to be more expensive as they fall under patent protection. For example, when the Hepatitis B (HepB) vaccine was developed, it remained unaffordable for many lower-income countries at a price of USD 30 per dose [23]. On the contrary, some vaccines are still very expensive, which includes Pertussive vaccines and is often combined with diphtheria and tetanus to produce Diphtheria, Tetanus whole-cell Pertussis (DTwP) and Diphtheria Tetanus Pertussis (DTaP), respectively. 

China is the world’s largest vaccine producer, accounting for the manufacturing of an estimated 700 million doses of vaccine annually [24,25]. In China, vaccines are provided to the population by government’s EPI, free of cost to the children up to 14 years of age [26]. The EPI was launched in China in 1978. It currently protects children from 12 vaccine-preventable diseases (VPDs): diphtheria, hepatitis A, hepatitis B, Japanese encephalitis, measles, meningococcal meningitis, mumps, pertussis, polio, rubella, tetanus and tuberculosis [26]. These vaccines are purchased by the government and are termed as Category 1 vaccines, while Category 2 (private sector) vaccines include Hemophilus influenzae type b vaccine (Hib), rabies vaccine, and influenza vaccine (InfV), HPV and rotavirus vaccines and are termed as optional vaccines [16,27]. It was observed that prices of Category 1 vaccines in China and some domestically produced Category 2 vaccines in China, such as seasonal influenza, Hib, Var, and ORV, were lower than prices of similar vaccines in the US, Europe, and UNICEF [16]. This could be due to the offering of lesser incentives secondary to the low prices to the manufacturers, which leads to the small investments in production and technology development for other combination vaccines at a domestic level. Furthermore, low cost vaccines are preferred, as they give freedom to vaccinate a large cohort of children for a vaccination budget [28]. On the contrary, some Category 2 vaccines are of high prices due to their small demand volumes, not meeting the economies of scale, when compared to the low-priced vaccines. Therefore, manufacturers of Category 2 vaccines earn profit from high prices, which supports the market survival and innovation for novel vaccines research and development, as can be seen from the China and India [29]. India and China are not investing in the development of new vaccines however, they are expanding their domestic manufacturing of vaccines [30,31].

Cervical cancer (CC) is the second most common female cancer worldwide, caused by Human Papilloma Virus, mainly affecting women aged between 35 and 55, with about 60,000 new cases in Europe each year [32,33]. The availability of prophylactic human papillomavirus (HPV) vaccines has provided an alternative for primary prevention of cervical cancer and other HPV-associated diseases [34]. The bivalent and quadrivalent HPV vaccines are responsible for the prevention of about 70–80% of cervical cancer and most cases of genital warts, while nonavalent vaccine prevents the remaining 20% of cervical cancer cases [35].

The bivalent and quadrivalent vaccines have been already licensed in over 100 countries and since 2012, HPV vaccines had become an important part of national immunization programs of at least 40 countries including Australia, Canada, the United Kingdom and United States and 22 countries from European region [32]. It was found that the total HPV vaccine sales for the manufacturers of both bivalent and quadrivalent vaccines amounted to UDD 14.1 billion in 2006–14 making a total fivefold return of investment to manufacturers [15]. 

In 2018, HPV vaccines market size was valued at USD 2.23 billion [35], which could reach up to USD 3.5 billion in 2025, estimating a larger share of revenues coming from the Asia Pacific region [36]. About 69% of the global market share of vaccines is contributed by high-income countries and upper middle-income countries, while low middle-income and low-income countries accounted for of 18 and 13% of shares, respectively. The estimated market share based on the volume of vaccine is such as that about 52% of volume is covered by tetravalent HPV vaccines, 25% by nonavalent, and 23 by bivalent HPV vaccines [34].

At the time of the launch, the first HPV vaccines were by far the most expensive of all available vaccines in the market [33], having a cost of USD 120.11 (EUR 100) per dose in most European countries [37]. However, with the passage of time, a decline in the price of HPV vaccines was observed. This was probably due to maturation in price. The per dose price of HPV vaccine funded by PAHO-RF decreased from USD 32.00 in 2010 to USD 13.50 in 2013 and then to USD 8.50 in 2015 [11]. 

The reasons of a drop in vaccine prices can be many, such as in the last few years, the prices of HPV vaccines have reduced significantly, for instance in Netherlands [38], where price per dose for each HPV vaccine was found to be USD 24.02 (EUR 20) and in Italy, it was USD 36.03 (EUR 30) [39]. The price drop was due to competitive tendering of prices in the case of HPV vaccines [40]. Another factor associated with the price decrease could be the involvement of organizations such as the GAVI alliance, the World Health Organization, the UNICEF, the World Bank and the Bill & Melinda Gates Foundation, and the PAHO-RF [41]. For instance, in 2000, the GAVI was established to help children worldwide, to improve the access to vaccines and support the vaccinations in 40 countries for 30 million girls by 2020 [42]. It has improved the affordability of vaccines, particularly for low-income countries [21]. This also further supports the view that GAVI-funded vaccines encourage more suppliers into the market and more competition, thus helping to lower the price of some vaccines [43].

It was observed that the affordable prices of vaccines can be achieved through tender procedures. This is because vaccine pricing is affected by contract volume and its duration, country, per capita GDP and the number of offers received [18]. This could also be explained by economic theory which suggests that with higher purchasing power on the demand side, the price tendering may achieve significant savings [44]. 

Spain, Italy and the US have also encouraged suppliers to stay in market by allowing multiple winners per tender or by using multiple tender lots, thus improving the sustainability of the vaccine ecosystem [45]. It was also noted that the tendering increases the price transparency, as the information is publicly available, and discounts are visible to different contracting authorities [33]. On the contrary, if low prices were achieved through tenders, it could possibly force some manufacturers out of market. This is because highly centralized and long duration tenders are maintained in the case of vaccines procurement [40,46]. Alternatively, short-term tenders, flexible procurement system and appropriate delivery timelines could help vaccines suppliers to remain in the market [45]. Increased per capita Gross Domestic Product (GDP) of the country was found to be directly linked with the increased tender prices for vaccines. This is evident from the WHO’s V3P (Product, Price, Procurement) project that shows vaccine prices vary widely region-wise and are positively linked with the country’s per capita income level [47]. For instance, in countries supported by GAVI, the maximum price for a vaccine type is six times higher than the minimum price reported in the same category in the countries without GAVI support. However, in high-income countries, the maximum price for a vaccine is almost 30 times higher. In middle-income countries, which are not supported by the Gavi, the maximum price for a vaccine type is 14 times higher creating a huge price differential. Other factors impacting on pricing include country’s income, quantity of purchased vaccines, purchasing method, duration of contract and the payment method used [2,48].

### Limitations of the Review

Whilst this systematic review highlights the pricing trend of vaccines from US, China and Europe, the review inherits several limitations. Firstly, as defined in inclusion criteria, studies published only in English language were included and those published in other languages were not included in this review. Despite this limitation, the review provides important insights into the pricing of vaccines and factors governing changes in pricing trend.

## 5. Conclusions

This systematic review summarizes recent original research on the topic of vaccine pricing. Most of this is related to price changes in childhood vaccines and HPV vaccines. It was observed that recently introduced vaccines were expensive, primarily due to the use of new and better technologies in the vaccines research, development, and production. The prices of vaccines were found to be decreased, if GAVI or other alliances were involved. It can be concluded from this review that affordable vaccine prices can be achieved through the involvement of a third party such as WHO, GAVI, UNICEF, PAHO-RF and procurement of vaccines through tender procedures, provided market failure is avoided.

## Figures and Tables

**Table 1:** Databases and keywords used in search strategies.

Database	Search Strategies
**PubMed**	(((((((((((((((((((((("vaccin"[Supplementary Concept] OR "vaccin"[All Fields]) OR "vaccination"[MeSH Terms]) OR "vaccination"[All Fields]) OR "vaccinable"[All Fields]) OR "vaccinal"[All Fields]) OR "vaccinate"[All Fields]) OR "vaccinated"[All Fields]) OR "vaccinates"[All Fields]) OR "vaccinating"[All Fields]) OR "vaccinations"[All Fields]) OR "vaccination s"[All Fields]) OR "vaccinator"[All Fields]) OR "vaccinators"[All Fields]) OR "vaccine s"[All Fields]) OR "vaccined"[All Fields]) OR "vaccines"[MeSH Terms]) OR "vaccines"[All Fields]) OR "vaccine"[All Fields]) OR "vaccins"[All Fields]) OR ((((((((((((((((((("vaccin"[Supplementary Concept] OR "vaccin"[All Fields]) OR "vaccination"[MeSH Terms]) OR "vaccination"[All Fields]) OR "vaccinable"[All Fields]) OR "vaccinal"[All Fields]) OR "vaccinate"[All Fields]) OR "vaccinated"[All Fields]) OR "vaccinates"[All Fields]) OR "vaccinating"[All Fields]) OR "vaccinations"[All Fields]) OR "vaccination s"[All Fields]) OR "vaccinator"[All Fields]) OR "vaccinators"[All Fields]) OR "vaccine s"[All Fields]) OR "vaccined"[All Fields]) OR "vaccines"[MeSH Terms]) OR "vaccines"[All Fields]) OR "vaccine"[All Fields]) OR "vaccins"[All Fields])) AND ((((((((("commerce"[MeSH Terms] OR "commerce"[All Fields]) OR "price"[All Fields]) OR "prices"[All Fields]) OR "costs and cost analysis"[MeSH Terms]) OR (("costs"[All Fields] AND "cost"[All Fields]) AND "analysis"[All Fields])) OR "costs and cost analysis"[All Fields]) OR "pricing"[All Fields]) OR "priced"[All Fields]) OR "pricings"[All Fields])) OR ((((((((("commerce"[MeSH Terms] OR "commerce"[All Fields]) OR "price"[All Fields]) OR "prices"[All Fields]) OR "costs and cost analysis"[MeSH Terms]) OR (("costs"[All Fields] AND "cost"[All Fields]) AND "analysis"[All Fields])) OR "costs and cost analysis"[All Fields]) OR "pricing"[All Fields]) OR "priced"[All Fields]) OR "pricings"[All Fields])) OR ((((((((("commerce"[MeSH Terms] OR "commerce"[All Fields]) OR "price"[All Fields]) OR "prices"[All Fields]) OR "costs and cost analysis"[MeSH Terms]) OR (("costs"[All Fields] AND "cost"[All Fields]) AND "analysis"[All Fields])) OR "costs and cost analysis"[All Fields]) OR "pricing"[All Fields]) OR "priced"[All Fields]) OR "pricings"[All Fields])
**Google Scholar**	vaccine OR vaccines AND price OR prices OR pricing
**Scopus**	vaccine OR vaccines AND price OR prices OR pricing AND (LIMIT-TO (PUBYEAR, 2020) OR LIMIT-TO (PUBYEAR, 2019) OR LIMIT-TO (PUBYEAR, 2018) OR LIMIT-TO (PUBYEAR, 2017) OR LIMIT-TO (PUBYEAR, 2016) OR LIMIT-TO (PUBYEAR, 2015)) AND (LIMIT-TO (DOCTYPE, "ar")) AND (LIMIT-TO (LANGUAGE, "English")
**Science Direct**	vaccine Or vaccines AND price OR prices OR pricing
**Springer Link**	vaccine Or vaccines AND price OR prices OR pricing

**Table 2:** Characteristics of studies selected.

Author and Year	Country	Vaccines Covered	Aims/Objectives of the Study	Key Findings
Chen et al., 2017 [15]	USA	Childhood vaccines	To explain the price changes among publicly purchased childhood vaccines recommended for routine vaccination	Recently introduced vaccines have higher prices Competition in the supply side of the vaccine market
Zheng et al., 2018 [16]	China	“National Immunization Program vaccine” and “Category 2 vaccine	To analyze selected aspects of vaccines and immunization in China and report the history, immunization policies, classification, supply, and price of vaccines in comparison with selected high- and middle-income countries	Procurement prices of EPI-substitute vaccines were higher than for their EPI equivalents. Category 1 vaccine prices were similar to UNICEF prices but lower than US and European prices for same vaccines. Prices of domestically produced Category 2 vaccines were lower than prices of similar vaccines in the US, Europe, and UNICEF. PCV7 and DTaP-IPV/Hib were higher in prices than similar vaccines in the US, Europe and UNICEF
Herlihy et al., 2016 [17]	Not applicable	Human Papillomavirus (HPV) vaccines	To examine the effect of six alternative pricing scenarios on the distribution of benefits obtained from HPV vaccines	Economic returns were five times of the original investment received by the manufacturers for HPV vaccine development High-income countries earned the greatest economic surplus Subsidizing vaccine prices in low- and middle-income countries could both reduce financial barriers to vaccine adoption
Qendri et al., 2019 [18]	Europe	Human Papillomavirus (HPV) vaccines	To analyze tender procedures for the HPV vaccines in Europe and to identify variables that are associated with HPV vaccine pricing in the different tender-based settings	HPV vaccine procurement is widely used across Europe. The tender-based prices resulted in the four times decline in vaccine prices as compared to the list prices proved tendering as an efficient cost-containment strategy.

**Table 3:** Vaccine programs, groups and prices.

Vaccines Program	Country	Vaccine Types	Vaccine Prices
Vaccine For Children	USA	Diphtheria, tetanus and pertussis (DTP) Hepatitis A (Hep A) Hepatitis B (Hep B) Hemophilus influenza type b (Hib) Human papillomavirus (HPV) Meningococcal Measles–mumps–rubella (MMR) Pneumococcal (Pneumo) Rotavirus (Rota) Varicella (Var)	USD 11 for price-capped vaccines USD 41 for combination vaccines
National HPV vaccination	Not applicable	Human Papillomavirus Vaccines-HPV	Not applicable
Expanded Program on Immunization	China	Pneumococcal Pneumonia Pneumonia and Meningitis Oral Rabies Vaccine (ORV) Varicella (Var) Diarrhea Brucellosis Seasonal influenza, Pestis Tick-bone Encephalitis (TBE) Typhoid fever Cholera Yellow fever (YF) Oral Poliovirus vaccine (OPV) Meningococcal meningitis Hepatitis (Hep) Tuberculosis Diphtheria, Tetanus, Pertussis (DTP) Japanese encephalitis (JEV-I) Leptospirosis (Leptospira) Measles, Mumps, Rubella (MMR) Anthrax (Anthrax) Hemorrhagic fever with renal syndrome (HFRS) Pandemic influenza (InfV) Enterovirus (EV71) Human papillomavirus (HPV)	Category 1: USD 0.1–5.7 Category 2: USD 2.4–102.9
National immunization programme	Europe	Human Papilloma Virus Divalent (2- valent) Quadrivalent (4-valent) Nonavalent (9- valent)	First-generation HPV vaccines from USD 122.27 to USD 34.11 (2007-2017), 9-valent vaccine in 2016–2017 was USD 59. Unit prices were, respectively, USD 9 and USD 41.31 higher for the 4-valent and 9-valent vaccines than for the 2-valent vaccine.
